# Effects of Apilarnil on Type 2 Diabetes-Induced IRS-1/PI3K/Akt Mediated Insulin Resistance in Male Rats

**DOI:** 10.1021/acsomega.5c02662

**Published:** 2025-06-03

**Authors:** Fatma Karagözoğlu, Emre Şahin, Şule Melek, Saliha Bediz Şahin, Recep Hakkı Koca, Hayati Yüksel, Alper Güngören

**Affiliations:** † Department of Animal Nutrition and Nutritional Diseases, Faculty of Veterinary Medicine, 37508 Dokuz Eylül University, İzmir 35890, Turkey; ‡ Department of Animal Nutrition and Nutritional Diseases, Faculty of Veterinary Medicine, 162312 Bingol University, Bingol 12000, Turkey; § Department of Surgery, Faculty of Veterinary Medicine, Bingol University, Bingöl 12000, Turkey; ∥ Department of Microbiology, Faculty of Veterinary Medicine, 37504 Ankara University, Ankara 06070, Turkey; ⊥ Department of Reproduction and Artificial Insemination, Faculty of Veterinary Medicine, Bingol University, Bingol 12000, Turkey; # Department of Pathology, Faculty of Veterinary Medicine, Bingol University, Bingol 12000, Turkey; ¶ Department of Food Hygiene and Technology, Faculty of Veterinary Medicine, 187466 Kastamonu University, Kastamonu 37150, Turkey

## Abstract

Diabetes is one of the most important chronic and metabolic health problems seen as an epidemic of the 21st century. The incidence of diabetes is gradually increasing, and different methods are used to treat it. Apitherapy products such as honey, propolis, royal jelly, and bee venom can be used as an alternative to treat diabetes and reduce symptoms. One of these apitherapy products is Apilarnil, which has not yet become popular. The hypolipidemic, hepatoprotective, androgenic, anabolic, and immune system enhancing properties of Apilarnil indicate that it may be effective in the treatment of type 2 diabetes or in reducing the symptoms. The study aimed to determine the therapeutic effect of Apilarnil on insulin resistance and the possible underlying mechanism. In the study, 40 8 week-old male Wistar albino rats were used. The rats were divided into 5 groups as Control, Diabetes, Diabetes + Api1 (125 mg/kg/day lyophilized Apilarnil), Diabetes + Api2 (250 mg/kg/day lyophilized Apilarnil) and Diabetes + Api3 (500 mg/kg/day lyophilized Apilarnil). Each group consisted of 8 rats. Apilarnil doses were administered by oral gavage with 1 mL of distilled water, 5 days in a week, during the last 6 weeks. The levels of Nrf2, NF-κB, TNF-α, total IRS-1, p-IRS-1, PI3K p85, total Akt, p-Akt, IL-1B, and GLUT4 proteins were determined in target tissues by Western blotting. Alanine aminotransferase (ALT), aspartate aminotransferase (AST), high-density lipoprotein (HDL), low-density lipoprotein (LDL), total cholesterol (TC), and triglyceride (TG) levels were detected in the serum. Serum superoxide dismutase (SOD), catalase (CAT), glutathione peroxidase (GSH-Px), malondialdehyde (MDA), and insulin levels were determined by the ELISA method. According to the data obtained in the study, the application of Apilarnil, compared to the diabetes group, balanced some findings, but did not demonstrate a fully antidiabetic effect.

## Graphical abstract

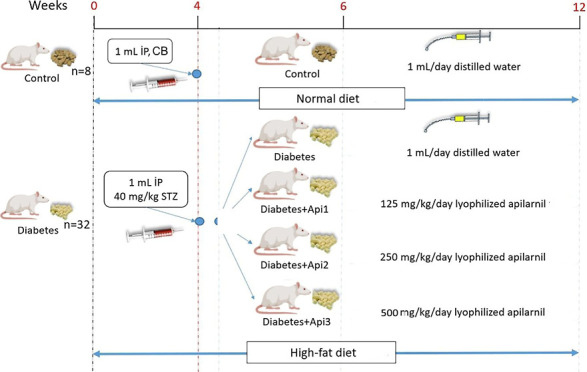

## 1 Introduction

Type 2 diabetes mellitus (T2DM) is a chronic and metabolic disease characterized by hyperglycemia, insulin resistance, and pancreatic β-cell dysfunction caused by metabolic disorders in the endocrine system.^1,2^ According to the Global Diabetes Alliance, there are 537 million adults with diabetes. This number is projected to rise to 643 million by 2030 and 780 million by 2045.^3^ Additionally, diabetes has become one of the top 10 global causes of mortality.^4^ Bommer et al.^5^ reported that the global economic cost of diabetes in 2015 was 1.3 trillion US dollars, and they predict that this figure will rapidly increase to 2.1 trillion US dollars by 2030. Diabetes is one of the most common metabolic diseases not only in humans but also in cats (type 2) and dogs (type 1).^6^

Persistent hyperglycemia in diabetes plays a critical role in developing and progressing diabetes-associated complications through the induction of proinflammatory cytokines, adipocytokines, and reactive oxygen species.^7^ In rats, insulin resistance develops in peripheral tissues following high-fat diet (HFD) feeding, and low-dose streptozotocin (STZ) injection can induce a mild impairment in pancreatic insulin production, leading to type 2 diabetes.^8^ HFD, which can cause a decrease in the total number of insulin receptors without receptor insufficiency,^9^ can reduce tyrosine phosphorylation by reducing the amount of insulin-mediated stimulation of insulin receptor substrates (IRS).^10^ IRS-1, together with glucose transporter type 4 (GLUT4), may play an important role in preventing the development of insulin resistance in muscle, fat and heart tissues.^11^ Decreased phosphorylation of IRS-1 decreases the activity of the phosphatidylinositol 3-kinase (PI3K)/protein kinase B (Akt) pathway. Inhibition of the IRS-1/PI3K/Akt pathway disrupts the regulation of metabolic functions such as glucose transport, adipogenesis, glycogen synthesis, and protein synthesis.^11,12^ Therefore, type 2 diabetes disrupts glycogen synthesis by affecting the IRS-1/PI3K/Akt pathway in the liver, and causes insulin resistance by affecting the IRS-1/PI3K/Akt/GLUT4 pathway in peripheral tissues. Insulin resistance leads to hyperglycemia and reactive oxygen species, which cause an increase in the severity of oxidative stress.^13^

Disorders that may occur as a result of type 2 diabetes can be reduced or treated through physical activity, nutritional regimen correction, functional nutrition, and some medical practices.^14,15^ Therefore, there is a need to develop new preventive and/or therapeutic strategies to combat Type 2 diabetes.

The number of alternative methods that can be used as supportive approaches in treating or preventing diabetes is steadily increasing. For this purpose, apitherapy products such as honey,^16^ propolis,^17^ royal jelly,^18^ and bee venom,^19^ which hold an important place among functional foods, can be used as alternative options for the treatment of diabetes and the alleviation of its symptoms. One of these bee products is Apilarnil, which has not yet gained popularity.^20^ Apilarnil is a natural bee product obtained from 3 to 7 day-old male bee larvae.^21^ Apilarnil is a highly concentrated nutritional substance containing carbohydrates, fats, proteins, minerals, vitamins, amino acids, polyphenols, hormones, unsaturated compounds (decanoic acids and sulfhydryl compounds), and sugars.^21–23^ The hypolipidemic, hepatoprotective, androgenic, anabolic and immune system enhancing properties of Apilarnil indicate that it may be effective in the treatment of type 2 diabetes or in reducing its symptoms.^24^ This study aimed to investigate the effects of Apilarnil administered to type 2 diabetic (HFD and STZ-induced) rats on IRS-1/PI3K/Akt/GLUT4 mediated insulin resistance and antioxidant enzymes in serum.

## 2 Materials and Methods

### 2.1 Animal Experimental Procedures

The animal experiments used to develop the Type 2 diabetes model were approved by the Bingol University Local Ethics Committee for Animal Experiments, decision number 06/02. Forty Wistar albino male rats weighing 180–220 g, and 8 weeks old, were provided. The number of animals (*N* = 40) was determined by power analysis using the G*Power program (Version 3.1.7).^25,26^ Therefore, 8 rats were used in each group to measure the blood glucose levels, based on the population number, with an α error (type 1 error) of 0.05, a confidence interval 95% power (1-β) and an effect size of 0.75.^27^ The experimental phase of the study was conducted in a room with temperature set at 22–24 °C ± 2 °C and humidity set at 55% ± 5%. The air taken in and out was filtered. The light/dark cycle of the experimental animal rooms were set as 12 h/12 h. The rats were housed in a total of 10 polypropylene cages, with 4 animals per cage (50 × 30 × 30 cm). Throughout the study, all efforts were made to minimize animal suffering and reduce stress. Animals were monitored daily for signs of pain, distress, or abnormal behavior. Humane end points, such as significant weight loss (≥20%), severe lethargy, or inability to access food and water, were predefined and would have prompted immediate euthanasia if observed. No animals reached these end points during the study period. In the study, lyophilized Apilarnil (Harşena Apitherapy Products) supplied by a commercial company was used.

The experiment lasted 14 weeks, including 2 weeks for the animals to adapt to the environment and 12 weeks for the trial period. Control rats (*n* = 8) were fed a normal diet (3219 Metabolic Energy kcal/kg and 22% crude protein) for 12 weeks and injected with citrate buffer (CB; 1 mL intraperitoneally) at the fourth week. Diabetic rats (*n* = 32) were fed HFD (4498 Metabolic Energy kcal/kg and 22% crude protein) with 53% of calories from fat for 12 weeks, followed by an STZ injection (40 mg/kg intraperitoneally, U-9889, Santa Cruz) at the fourth week. Three days after STZ injections, blood glucose levels were measured and animals with ≥200 mg/dL were considered Type 2 diabetic. Diabetic rats (*n* = 32) were divided into 4 groups by complete randomization method after the fifth week. Groups;

I **Control**: 1 mL/day distilled water was administered by oral gavage, 5 days a week, for the last 6 weeks.
II **Diabetes**: 1 mL/day distilled water was administered by oral gavage, 5 days a week, for the last 6 weeks.
III **Diabetes** + **Api1**: 125 mg/kg/day lyophilized Apilarnil was administered by oral gavage in a volume of 1 mL, 5 days a week, for the last 6 weeks.
IV **Diabetes** + **Api2**: 250 mg/kg/day lyophilized Apilarnil was administered by oral gavage in a volume of 1 mL, 5 days a week, for the last 6 weeks.
V **Diabetes** + **Api3**: 500 mg/kg/day lyophilized Apilarnil was administered by oral gavage in a volume of 1 mL, 5 days a week, for the last 6 weeks.

### 2.2 Estimation of Feed Consumption and Body Weight

The animals’ feed consumption was calculated on a group basis. Body weight measurements were made at the beginning (day 0), second, fourth, sixth, eighth, 10th, and 12th weeks.

### 2.3 Oral Glucose Tolerance Test

The oral glucose tolerance test (OGTT) was performed for each group the day before the end of the study. Glucose (2 g/kg) was given orally to rats that fasted for 12 h between 20:00 and 08:00 the next day. Blood samples were taken from the tail vein at 0, 30, 60, and 120 min, and glucose levels were measured. The area under the glucose curve (AUC) was determined and compared for each group. AUC = 0.25 × *A* + 0.5 × *B* + 0.75 × *C* + 0.5 × *D* (*A*, *B*, *C* and *D* are blood glucose levels at 0, 30, 60, and 120 min, respectively) was calculated with the formula.

### 2.4 Blood Glucose Level, Insulin Resistance (HOMA-IR), Beta Cell Function (HOMA-β), Insulin Sensitivity Measurement

Blood glucose measurements were performed at baseline (day 0), 2, 4, 6, 8, 10, and 12 weeks. After the animals had fasted for 12 h, blood samples were taken from the tail vein using a commercial glucometer (Clever Chek TD-4231).

Insulin Resistance (HOMA-IR) was determined using the formula “fasting insulin (U/L) × fasting glucose (nmol/L)/22.5” based on the blood glucose and serum insulin levels measured on the last day of the trial.

TThe cutoff value of HOMA-IR was then determined by receiver operating characteristic (ROC) analysis in the control and diabetes groups. Rats above the determined HOMA-IR value were considered insulin-resistant.

Beta Cell Function (HOMA-β) was determined with the formula “[(20 × fasting insulin (U/L))/(fasting glucose (nmol/L)-3.5)”].

Insulin sensitivity was determined with the formula “1/(log­[fasting glucose (nmol/L)]) × log­[fasting insulin (U/L)]”.

### 2.5 Anesthesia, Necropsy, Collection of Blood and Tissue Samples

During the last 12 h of the experiment, the animals were fasted, weighed, and then euthanized. The rats were euthanized by decapitation and their blood samples were taken. Serum obtained from the blood samples was stored at −80 °C until analysis. In addition, the weights of visceral fat, liver and pancreas tissues were determined. The liver and muscle to be subjected to molecular analysis were placed in sealed bags and stored at −80 °C.

### 2.6 Serum Biochemistry Analyses

Serum aspartate aminotransferase (AST), alanine aminotransferase (ALT), low-density lipoprotein (LDL), high-density lipoprotein (HDL), total cholesterol (TC), and triglyceride (TG) levels were measured as specified in the user manual of commercial reagent kits.

### 2.7 Enzyme-Linked Immunosorbent Assay (ELISA) Test

The Sandwich ELISA method was performed using commercial kits. SOD, CAT, GSH-Px, MDA, and insulin standard antibody detection curves were prepared for each application. The amount of SOD, CAT, GSH-Px, MDA, and insulin in serum was determined using rough dilution multiples, and absorbances were measured using an (ELx808 Absorbance Microplate Reader).

### 2.8 Western Blot Analysis

The protein levels of Nrf2, NF-κB, TNF-α, total IRS-1, p-IRS-1 (phospho Y632), PI3K p85, total Akt, and p-Akt (phospho T308) in the liver and total IRS-1, p-IRS-1 (phospho Y632), PI3K p85, total Akt, p-Akt (phospho T308), and GLUT4 in the muscle were analyzed using the Mini-PROTEAN Tetra Vertical Electrophoresis Chamber (Biorad, Hercules, California, USA), the Trans-Blot Turbo Transfer System (Biorad, Hercules, California, USA), electrophoresis, and the Western blot system.^28^ The tissues were homogenized with the buffered solution prepared for homogenization, and the homogenates obtained were stored in a −80 °C deep freezer until analyzed. Protein samples in tissue homogenates were run on a 12% gel (acrylamide-bis­(acrylamide)) using SDS-PAGE, before these proteins were transferred to a nitrocellulose membrane using Western blotting. The interactions of primary and secondary antibodies were detected using the chromogenic method, resulting in the appearance of bands. The synthesis rates of the proteins were examined, and these bands’ intensities were subsequently analyzed densitometrically using a computer program (ImageJ; National Institute of Health, Bethesta, USA).

### 2.9 Statistical Analysis

Data are presented as mean ± standard error and *P* < 0.05 was considered significant. Statistical analyses were performed using the SPSS statistics program (22.0, Chicago, IL, USA), the GraphPad Prism for Windows program ver. 5.0 (GraphPad software Inc., SanDiego, CA, USA), and the R Studio program (RStudio, Version 3.6, Boston) programs. The Shapiro–Wilk test was used to determine the normality of data distributions, while the variance homogeneity of groups was determined by the Levene test. One-way analysis of variance followed by a Tukey test was performed to determine the differences between the groups.

## 3 Results

### 3.1 Feed Consumption, Body Weight Change

The effect of Apilarnil administration on body weight change and feed consumption in rats with diabetes induced by HFD and STZ administration was determined during the 12 week study (Figure 1A,B). The body weights of male rats in each group were measured seven times at the beginning (day 0), second, fourth, sixth, eighth, 10th and 12th weeks (Figure 1A). While no statistically significant difference was observed in the body weight of diabetic animals compared to the control group in the sixth and eighth weeks (*p* > 0.05), a nonsignificant decrease in their body weight was observed. There were significant decreases in the body weights of type 2 diabetic animals compared to the control group, especially in the 10th and 12th weeks of the study (*p* < 0.05). Feed consumption of animals was calculated on a group basis. Induced diabetes in rats increased feed consumption (Figure 1B, *p* < 0.05). Apilarnil application prevented polyphagia and decreased feed consumption (*p* < 0.05).

**Figure 1:**
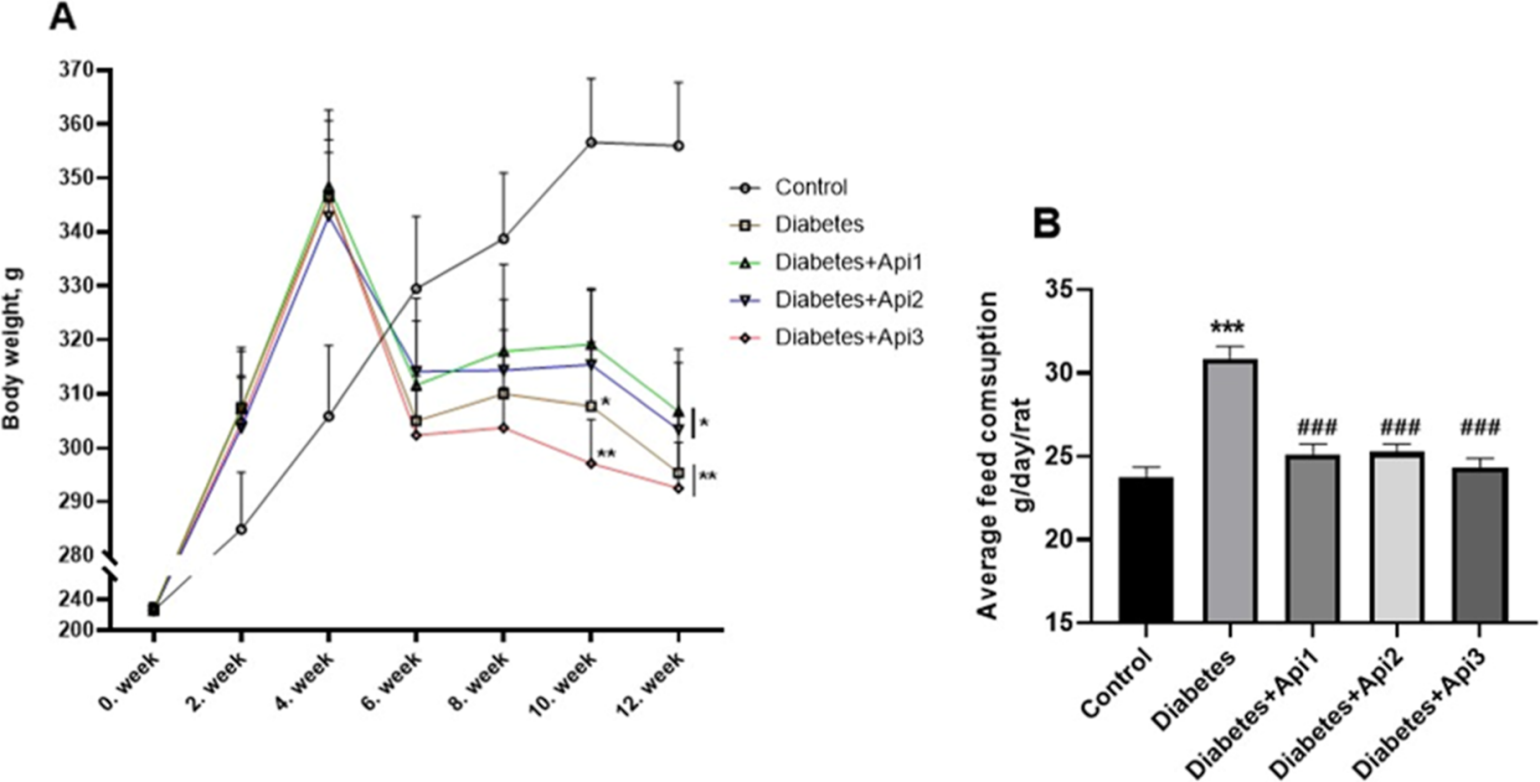
Effect of Apilarnil on body weight change (A) and feed consumption (B) in rats. *p < 0.05, **p < 0.01 and ***p < 0.001 indicate difference compared to control group, ###*p* < 0.001 indicate difference compared to diabetes group.

### 3.2 Oral Glucose Tolerance Test (OGTT)

When the diabetes groups were compared according to the area under the curve (AUC) levels obtained from the OGTT, it was found that the Diabetes group had the highest AUC level and the Diabetes + Api3 group had the lowest AUC level (Figure 2, *p* < 0.001).

**Figure 2:**
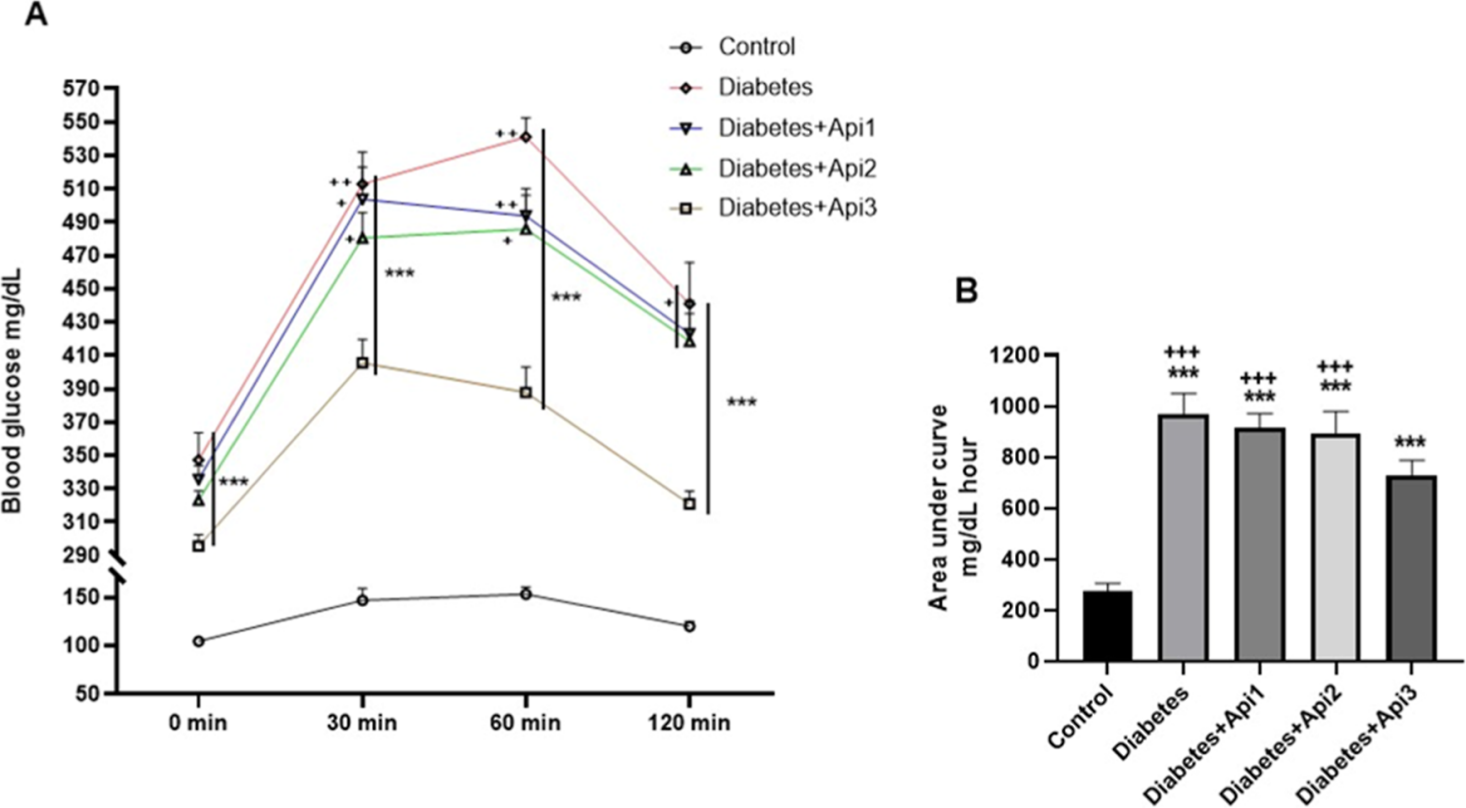
Effect of Apilarnil on glucose tolerance (A) and area under the curve (B) in rats. ***p < 0.001 indicates a difference compared to the control group, +*p* < 0.05. ++*p* < 0.01 and +++*p* < 0.001 indicate a difference compared to the Diabetes + Api3 group.

### 3.3 Blood Glucose Level, Insulin Resistance (HOMA-IR), Beta Cell Function (HOMA-β), Insulin Sensitivity

Blood glucose values of all animals used in the experiment were measured 7 times at intervals at the beginning of the experiment (day 0) and at the second, fourth, sixth, eighth, 10th and 12th weeks (Figure 3). In the type 2 diabetic rat model created with HFD and STZ administration, blood glucose levels showed a significant increase compared to the control group (Figure 3, *p* < 0.001). In the last week of the study, the blood glucose levels of the Diabetes + Api3 group decreased to 17.6% and 13.5% compared to the Diabetes and Diabetes + Api1 groups (*p* < 0.01 and *p* < 0.05, respectively). Administration of Apilarnil doses to rats in the diabetes group did not reduce blood glucose levels below 200 mg/dL.

**Figure 3:**
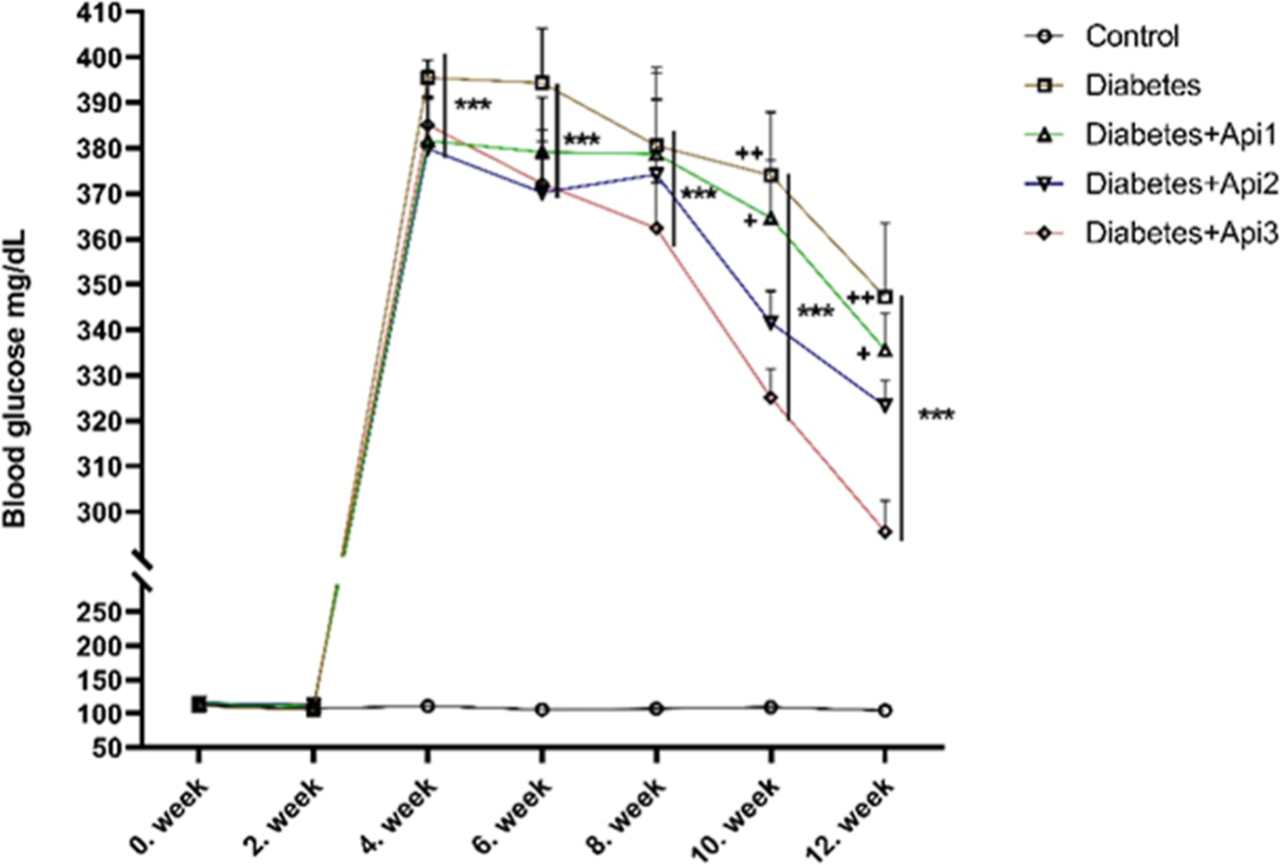
Effect of Apilarnil on blood glucose level (mg/dL) in rats. ***p < 0.001 indicates a difference compared to the control group, +*p* < 0.05 and ++*p* < 0.01 indicate a difference compared to the Diabetes + Api3 group.

In diabetic rats, serum insulin levels did not change compared to the control group (*p* > 0.05). In diabetic rats, insulin resistance (HOMA-IR index) increased (*p* < 0.0001), while beta cell function (HOMA-β index) and insulin sensitivity index (*p* < 0.0001) decreased compared to the control group (Figure 4).

**Figure 4:**
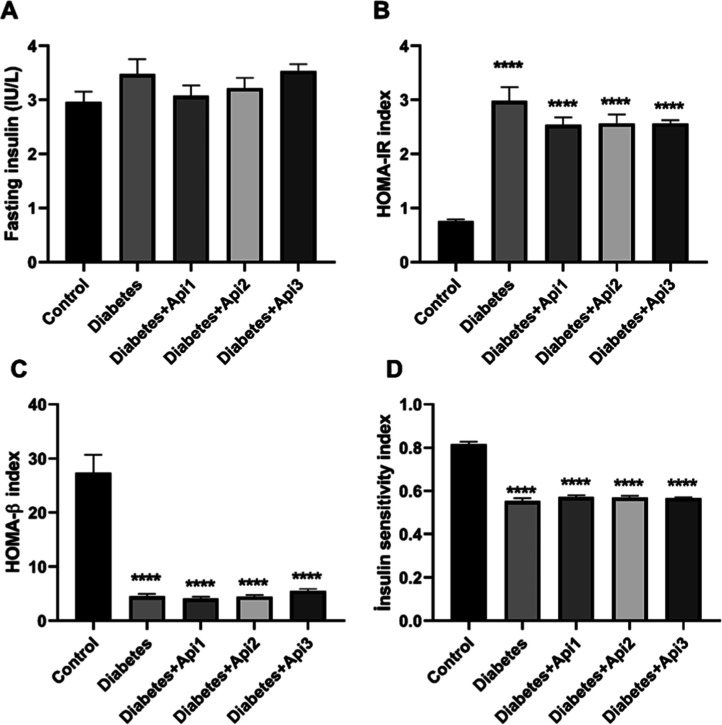
Effect of Apilarnil on serum fasting insulin level (A), HOMA-IR index (B), HOMA-β index (C) and insulin sensitivity index (D) in rats. ****p < 0.0001 indicates difference compared to the control group.

### 3.4 Weights of Tissues

Compared with the control group, no difference was observed in the liver weights of the diabetes groups (*p* > 0.05), while a significant decrease was detected in pancreas and visceral fat weights (Figure 5, *p* < 0.01 and *p* < 0.001).

**Figure 5:**
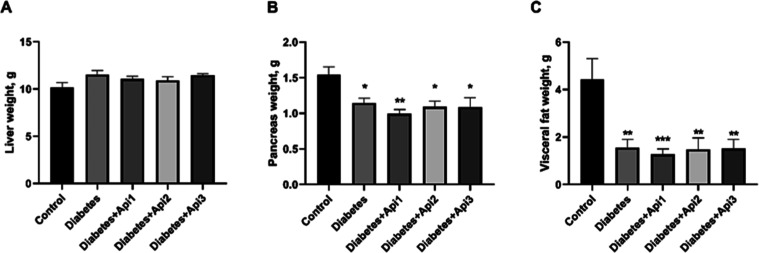
Effect of Apilarnil on liver (A), pancreas (B) and visceral fat (C) weights in rats. *p < 0.05, **p < 0.01 and ***p < 0.001 indicate differences compared to the control group.

### 3.5 Serum Biochemistry Analyses

Serum ALT and AST levels were significantly increased in animals with induced type 2 diabetes (Figure 6, *p* < 0.0001). Serum triglyceride (*p* < 0.0001), low-density lipoprotein (*p* < 0.001), and total cholesterol (*p* < 0.001) levels increased in diabetic animals, while high-density lipoprotein levels decreased (Figure 6, *p* < 0.05). No statistical difference was found between the control group and the Diabetes + Api1 group in terms of serum TG, LDL and TC levels (*p* > 0.05). In addition, Apilarnil administered at a dose of 125 mg/kg effectively reduced serum TG, LDL, and TC levels in the diabetes group (*p* < 0.01).

**Figure 6:**
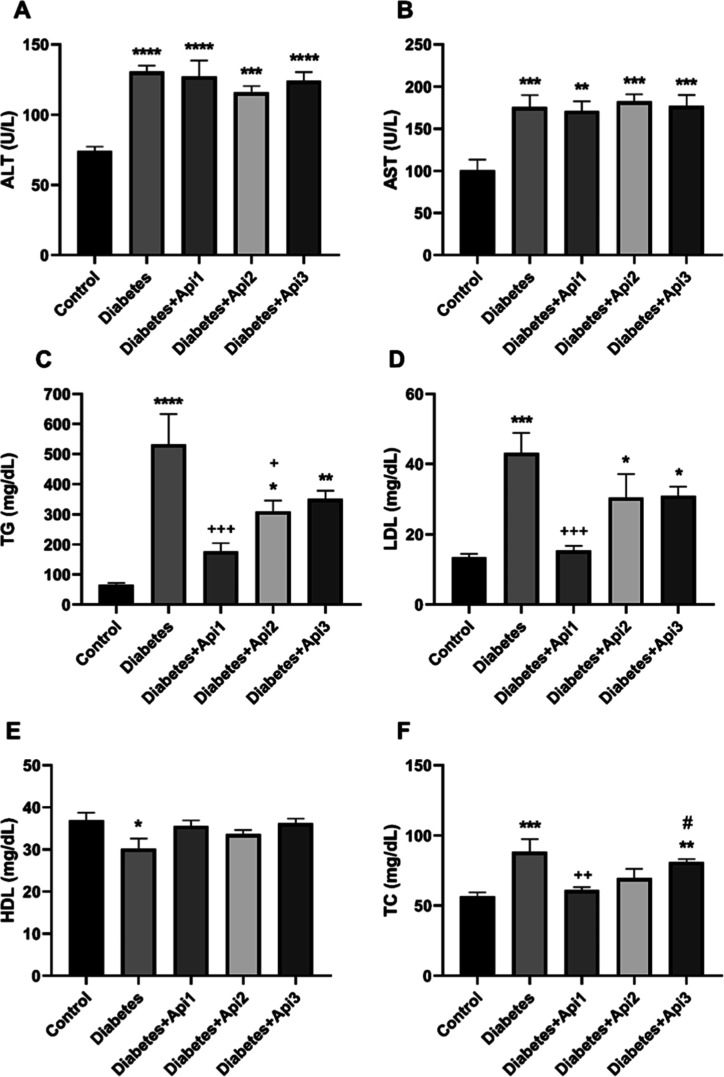
Effects of Apilarnil on serum alanine aminotransferase (ALT, A), aspartate aminotransferase (AST, B) triglyceride (TG, C), low-density lipoprotein (LDL, D), high-density lipoprotein (HDL, E) and total cholesterol (TC, F) levels in rats. *p < 0.05, **p < 0.01, ***p < 0.001 and ****p < 0.0001 Compared to the control group; +*p* < 0.05, ++*p* < 0.01, +++*p* < 0.001 Compared to the diabetes group; #*p* < 0.05 Compared to the diabetes + Api1 group.

### 3.6 Enzyme-Linked Immunosorbent Assay (ELISA) Test

It was found that the level of antioxidant enzymes (SOD, CAT and GSH-Px) in the serum showed a significant decrease in diabetic animals (Figure 7, *p* < 0.001). In contrast to antioxidant enzymes, the level of malondialdehyde (MDA), a lipid peroxidation marker in the serum, increased in diabetic animals (Figure 7, *p* < 0.0001).

**Figure 7:**
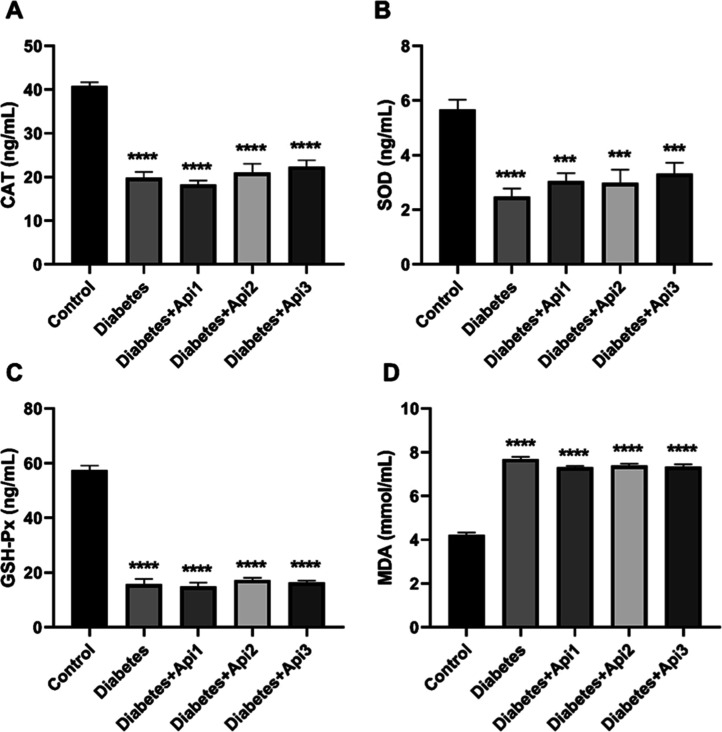
Effect of Apilarnil on serum catalase (CAT, A), superoxide dismutase (SOD, B), glutathione peroxidase (GSH-Px, C) and malondialdehyde (MDA, D) levels in rats. ***p < 0.001 and ****p < 0.0001 indicate differences compared to the control group.

### 3.7 Western Blot

Nrf2 (*p* < 0.0001), total IRS-1 (*p* < 0.01), p-IRS-1 (*p* < 0.0001), p-Akt (*p* < 0.0001) and PI3K p85 (*p* < 0.0001) proteins were significantly suppressed in the livers of type 2 diabetes-induced animals (Figure 8). On the other hand, NF-κB and TNF-α protein levels, which are important inflammatory markers, also increased in diabetic animals (*p* < 0.0001). However, different doses of Apilarnil treatments were not effective in normalizing the levels of these proteins in the liver.

**Figure 8:**
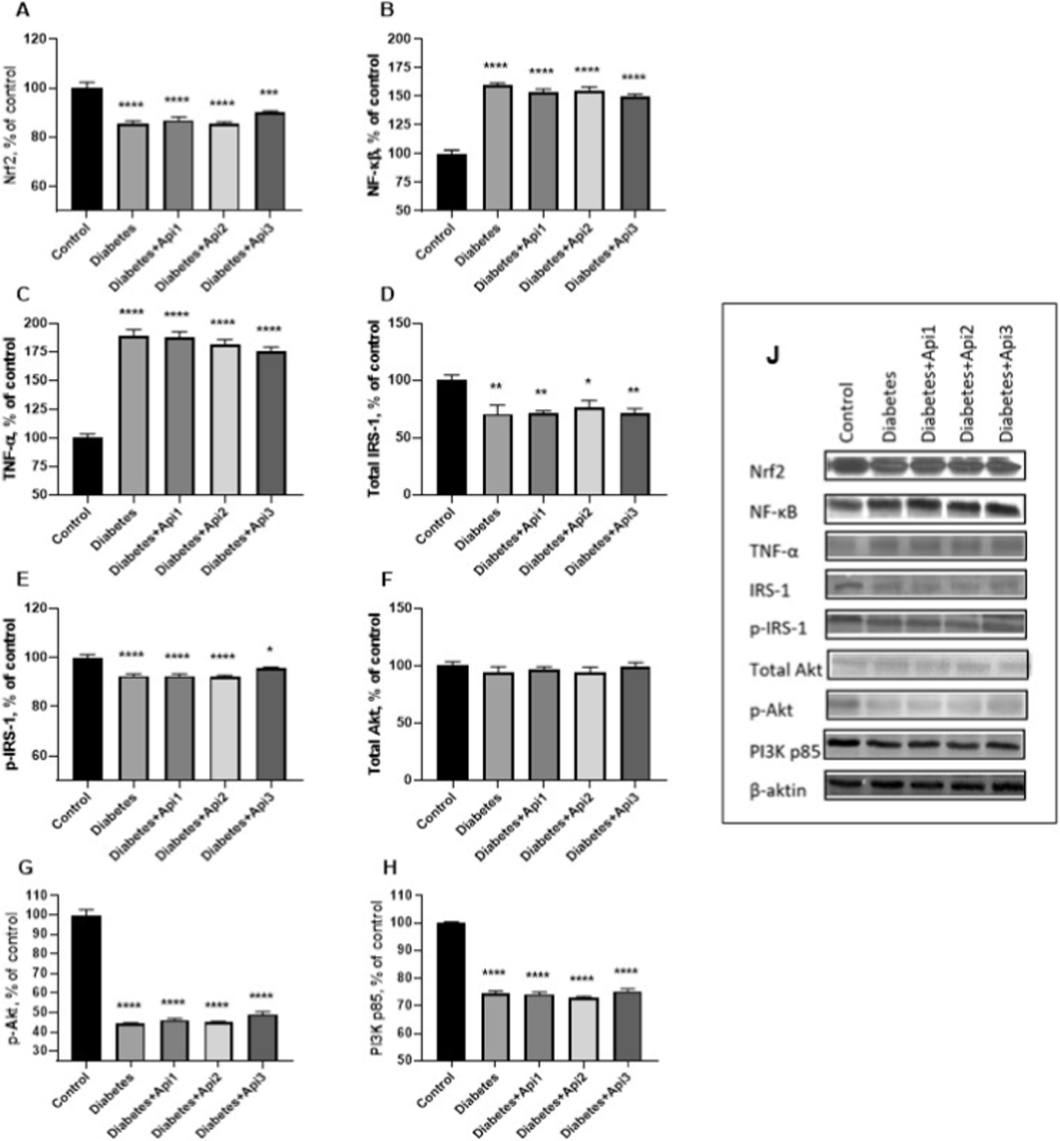
Effect of Apilarnil on liver Nrf2 (A), NF-κB (B), TNF-α (C), total IRS-1 (D), p-IRS-1 (E), total Akt (F), p-Akt (G), and PI3K p85 (H) protein expression levels (J) in rats. β-Actin was used as a loading control. Data are organized according to the percentage of the control group. *p < 0.05, **p < 0.01, ***p < 0.001 and ****p < 0.0001 indicate differences compared to the control group. The full blots of liver protein expression levels are presented in Figure S1 in the Supporting Information.

Total IRS-1, p-IRS-1, p-Akt, PI3K p85 and GLUT4 (*p* < 0.0001) proteins in muscle tissues of type 2 diabetes-induced animals were significantly decreased (*p* < 0.0001) (Figure 9). It was observed that Apilarnil application was insufficient to improve the level of target proteins in muscle tissue.

**Figure 9:**
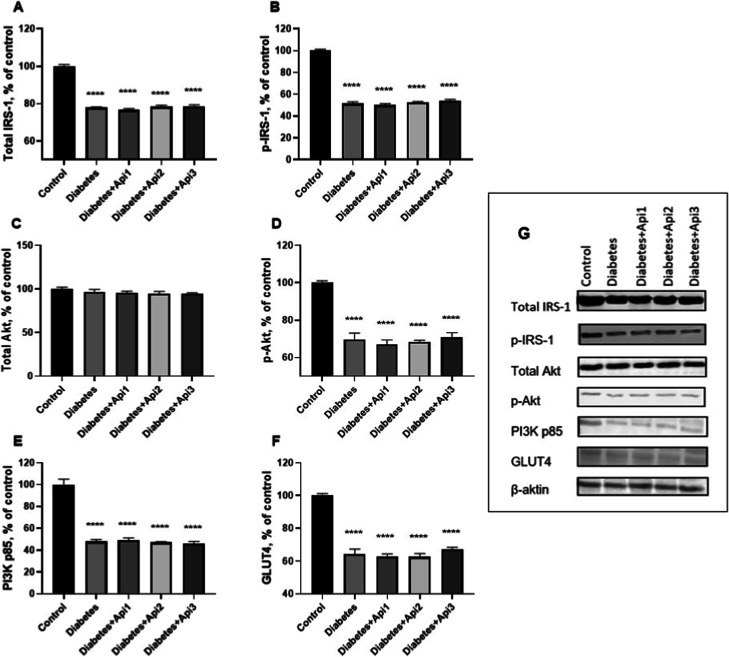
Effect of Apilarnil on muscle total IRS-1 (A), p-IRS-1 (B), total Akt (C), p-Akt (D), PI3K p85 (E), and GLUT4 (F) protein expression levels (G) in rats. β-Actin was used as a loading control. Data are organized according to the percentage of the control group. ****p < 0.0001 indicates difference compared to the control group. The full blots of muscle protein expression levels are presented in Figure S2 in the Supporting Information.

## 4 Discussion

The effect of Apilarnil administration on body weight change and feed intake in rats with diabetes induced by HFD and STZ administration was observed during the 12 week study. In general, chronic long-term feeding with a high-fat diet leads to an increase in body weight due to the accumulation of saturated fats in the body. However, following streptozotocin administration, uncontrolled hyperglycaemia results in weight loss in diabetic animals.^29^ In other words, diabetic rats may experience weight loss compared to control rats.^30^ This study is consistent with these findings. In our study, significant reductions in body weight were observed in type 2 diabetic animals compared to the control group, particularly in the 10th and 12th weeks of the study. However, it was determined that Apilarnil given to rats did not have any effect on preventing weight loss. Previous studies conducted on broilers have also shown that Apilarnil does not affect the body weight of animals.^31,32^ It has been reported that diabetes is associated with weight loss, polydipsia, polyphagia, and polyuria.^33^ In terms of feed consumption, diabetes induced in rats increased feed consumption. However, Apilarnil administration prevented the polyphagic state and reduced feed consumption.

The increased serum glucose level in the groups in which diabetes was induced with HFD and STZ administration shows a specific hyperglycemia profile for type 2 diabetes. In rats with diabetes induced by HFD and STZ, blood glucose values increased by approximately 3.7-fold compared to the control group. In the final week of the study, the blood glucose levels of the Diabetes + Api3 group decreased by 17.6% and 13.5% compared to the Diabetes and Diabetes + Api1 groups (*p* < 0.01 and *p* < 0.05, respectively). In a previous study conducted on broilers, the fact that Apilarnil (7.5 g/day) administration resulted in statistically significant decreases in blood glucose and serum total cholesterol levels supports these findings.^32^ However, the observation that the blood glucose levels of the rats in the Diabetes + Api3 group followed a high level (>200 mg/dL) indicates that the doses of apilarnin used in the current study did not produce a significant effect in diabetes. Similarly, based on the area under the curve (AUC) levels obtained from the oral glucose tolerance test (OGTT), it was found that the Diabetes group had the highest AUC level, whereas the Diabetes + Api3 group had the lowest AUC level.

When previous studies are examined, serum insulin levels in diabetic rats induced by HFD and STZ have been observed to decrease,^34^ increase^35^ or remain unchanged.^9^ Serum insulin level may not change in type 2 diabetes mellitus induced by HFD and low dose STZ compared to the control group as in this study. In diabetic animals, insulin resistance (HOMA-IR index) increased (*p* < 0.0001) due to increased serum glucose levels, while beta cell function (HOMA-β index) and insulin sensitivity index (*p* < 0.0001) decreased compared to the control group. None of the Apilarnil doses was effective in reducing the HOMA-IR index. In addition, the HOMA-β index and insulin sensitivity index were not increased by Apilarnil administration.

Pancreas and visceral fat weights are decreased in diabetic animals compared to control rats.^36,37^ The number and size of the islets are associated with pancreatic weight, and the reduction of both parameters may explain the significant reduction in pancreatic weight observed in the animals.^38^ In this study, pancreatic and visceral fat weights decreased after induction of diabetes. However, different doses of Apilarnil could not prevent this decrease. Regarding liver weight, increased dyslipidaemia, oxidative stress, and inflammation may contribute to weight gain in the diabetes group.^39^ In this study, although no significant difference in liver weight was detected among the groups when compared to the control group, a slight increase in liver weight was nevertheless observed.

Previous studies have shown that serum AST, ALT, TG, TC and LDL levels increase in consistent with type 2 diabetes, while HDL levels decrease.^30,40^ Altered metabolism due to insulin insufficiency/resistance in diabetes increases blood lipid levels (such as total cholesterol, triglyceride, LDL, VLDL).^41,42^ Impairment of fat metabolism leads to an influx of free fatty acids (FFA) from adipose tissue to other tissues, especially the liver, which results in increased synthesis and secretion of very low-density lipoprotein (VLDL). Increased VLDL levels regulate the low-density lipoprotein (LDL) receptor while decreasing LDL cholesterol particles and high-density lipoprotein (HDL) cholesterol levels, thus increasing peripheral TG concentration. This increases the fatty acid pool in hepatocytes, creating a vicious circle and leading to a further increase in VLDL production.^41,43,44^ Serum AST and ALT enzymes are examined as indicators of liver function. In the case of hyperglycemia, carbohydrate, protein, and lipid metabolism disorders, together with oxidative stress, negatively affect liver functions. Increased AST and ALT enzymes in diabetic rats are indicators of active liver damage. The increase in enzymes in diabetes is due to increased ketogenesis and gluconeogenesis.^30,45^ Apilarnil applications did not reduce elevated ALT and AST enzyme values. However, due to the hypolipidemic effect of Apilarnil,^46^ it has been concluded that serum lipid levels are more balanced compared to the Diabetic group. The presence of diverse fatty acids, particularly palmitoleic and oleic acids, supports Apilarnil’s potential in modulating lipid metabolism and insulin signaling.^47^ Additionally, we observed that Apilarnil’s hypolipidemic effect was not dose-dependent, suggesting that Apilarnil may exhibit hormetic effects on lipid metabolism. This hormetic effect might stem from the fatty acid composition of Apilarnil, particularly palmitic acid or conjugated linoleic acid (CLA). Lyophlised Apilarnil contains 21.1–24.2% lipids^47^ and 52.62% CLA (% of total fatty acids).^48^ Previously, studies have reported that insulin resistance in obese/diabetic mice is promoted by CLA nutrition, which inversely regulates leptin and adiponectin, known to either improve or worsen insulin sensitivity.^49^ Also, palmitic acid can contribute to aberrant insulin regulation of glucose metabolism in type 2 diabetes.^50^ Therefore, high doses of Apilarnil might have failed to improve dyslipidemic conditions in type 2 diabetic rats.

In diabetes, the activities of antioxidant enzymes such as catalase, superoxide dismutase, and glutathione peroxidase decrease, and oxidative damage may occur in tissues.^40^ Similarly, in the study, SOD, CAT, and GSH-Px levels in the serum of diabetic rats showed a significant decrease. In contrast, the level of MDA, a byproduct of lipid peroxidation increased. In diabetes, due to high oxidative stress caused by hyperglycemia, the activity of the antioxidant defense system decreases, and the production of free radicals increases.^16^ Despite the antioxidant activity,^51^ Apilarnil failed to enhance the activity of antioxidant enzymes in animals with induced type 2 diabetes. The antioxidant activity of Apilarnil is primarily attributed to its content of flavonoids, phenolic acids, and coenzyme Q10.^47^ However, a six-week period of Apilarnil administration may have negatively impacted its antioxidant properties in rats with type 2 diabetes.

Suppression of Nrf2 activity in type 2 diabetic rat models may lead to failure to coordinate genes showing antioxidant activity and a decrease in antioxidant capacity. This situation results in an increased release of key pro-inflammatory cytokines such as NF-κB, TNF-α, and IL-1β. In addition, levels of antioxidant enzymes such as CAT and SOD may decrease due to decreased Nrf2 activity.^52,53^ In this regard, similar to previous studies, in our study, it was observed that the levels of NF-κB^16,54^ and TNF-α ^54,55^ proteins increased in the liver tissues of animals due to inflammation induced by type 2 diabetes. Studies in nondiabetic animals have suggested that Apilarnil has hepatoprotective and anti-inflammatory properties.^56,57^ However, in this study, the effects of Apilarnil in diabetic animals were investigated and it was concluded that it did not have a healing effect on the inflammatory status in the liver at the molecular level. Apilarnil may have been ineffective in diabetic animals due to its high energy content and carbohydrates such as glucose and fructose,^21,58^ despite containing compounds that increase antioxidant activity.^51^

The IRS-1/PI3K/Akt pathway is one of the important targets in the treatment of diabetic animals. IRS-1, together with GLUT4, can play an important role in preventing the development of insulin resistance in muscle, fat, and heart tissues.^16^ After decreased phosphorylation of IRS-1, the activity of the PI3K/Akt pathway decreases. Inhibition of the IRS-1/PI3K/Akt pathway may disrupt the regulation of metabolic functions such as glucose transport, adipogenesis, glycogen synthesis, and protein synthesis.^11,59^ In type 2 diabetic animals, IRS-1 phosphorylation is reduced due to insulin resistance, and as a result, cellular membrane translocation of GLUT4 in peripheral tissues may be suppressed.^13^ IRS-1 and subsequent stimulation of GLUT4 may also be related to the PI3K/Akt pathway. In type 2 diabetes, the levels of PI3K p85 and p-Akt proteins are decreased in the liver, muscle, and visceral fat tissue.^60,61^ In this study, after inducing type 2 diabetes using HFD and STZ, there was a decrease in the levels of IRS-1, PI3K, and GLUT4 proteins in the liver and muscle tissue, as well as a suppression in the levels of phosphorylation of IRS-1 and Akt. However, no dose of Apilarnil positively or negatively affected the IRS-1/PI3K/Akt-mediated signaling pathway. Apilarnil may be ineffective on this pathway in diabetic animals due to its high energy content and carbohydrates such as glucose and fructose.^21,58^ Apilarnil (200 or 400 mg/kg dose) can recover the imatinib-induced PI3K/AKT/mTOR pathway in rats.^62^ However, when energy stress occurs, AMP-activated protein kinase (AMPK) may be triggered and anabolic activities could be prevented.^63^ AMPK phosphorylation may inhibit IRS-1/PI3K/Akt signaling due to energy depletion.^64^ Muscle glucose uptake and glycogen synthesis are significantly impaired in type 2 diabetic rats, which directly impacts energy availability.^65^ Notably, it includes kaempferol, quercetin, and apigenin 7-glucoside, with a total flavonoid content of 13.16 ± 0.94 mg/100 g. These flavonoids and polyphenols are known for AMPK activation.^47^ In the current study, this AMPK pathway might be suppressed by the IRS-1/PI3K/Akt-mediated signaling. On the other hand, the decrease in serum glucose and AUC values in the Diabetes + Api3 group compared to the diabetes group indicates that the effects of Apilarnil on type 2 diabetes might be mediated through different pathways.

In conclusion, although Apilarnil influenced several metabolic parameters such as serum glucose, TG, TC, and LDL in diabetic animals, it failed to elicit a significant antidiabetic effect via the IRS-1/PI3K/Akt signaling pathway. These results highlight the limited efficacy of Apilarnil as an antidiabetic agent. In addition, more comprehensive studies for more accurate and conscious use of Apilarnil may open new horizons in the field of diabetes. Understanding the mechanisms of action of Apilarnil may contribute to the development of appropriate strategies for alleviating various diseases or their complications.

## Supplementary Material



## Notes

Consists of the full blots of liver (Figure S1) and muscle (Figure S2) protein expression levels in rats (PDF)
