# Supplementary material for: Effects of Apilarnil on Type 2 Diabetes-Induced IRS-1/PI3K/Akt Mediated Insulin Resistance in Male Rats
Source: ACS Omega. 2025 Jun 3;10(23):25027–38. doi: 10.1021/acsomega.5c02662 (PMC12177753; doi:10.1021/acsomega.5c02662)
Supplement: Supplementary file 1 [file ao5c02662_si_001.pdf]

Supporting Information

Effects of Apilarnil on Type 2 Diabetes-Induced IRS-1/PI3K/Akt Mediated Insulin Resistance in Male Rats

Fatma Karagözoğlu,[*,1] Emre Şahin[2], Şule Melek[3], Saliha Bediz Şahin[4], Recep Hakkı Koca[5], Hayati Yüksel[6], Alper Güngören[7]

[1]Dokuz Eylül University, Faculty of Veterinary Medicine, Department of Animal Nutrition and Nutritional Diseases, İzmir, 35890, Turkey

[2]Bingol University, Faculty of Veterinary Medicine, Department of Animal Nutrition and Nutritional Diseases, Bingol, 12000, Turkey

[3]Bingol University, Faculty of Veterinary Medicine, Department of Surgery, Bingöl, 12000, Turkey

[4]Ankara University, Faculty of Veterinary Medicine, Department of Microbiology, Ankara, 06070, Turkey

[5]Bingol University, Faculty of Veterinary Medicine, Department of Reproduction and Artificial Insemination, Bingol, 12000, Turkey

[6]Bingol University, Faculty of Veterinary Medicine, Department of Pathology, Bingol, 12000, Turkey

[7]Kastamonu University, Faculty of Veterinary Medicine, Department of Food Hygiene and Technology, Kastamonu, 37150, Turkey

*Corresponding Authors: fatma.karagozoglu@deu.edu.tr

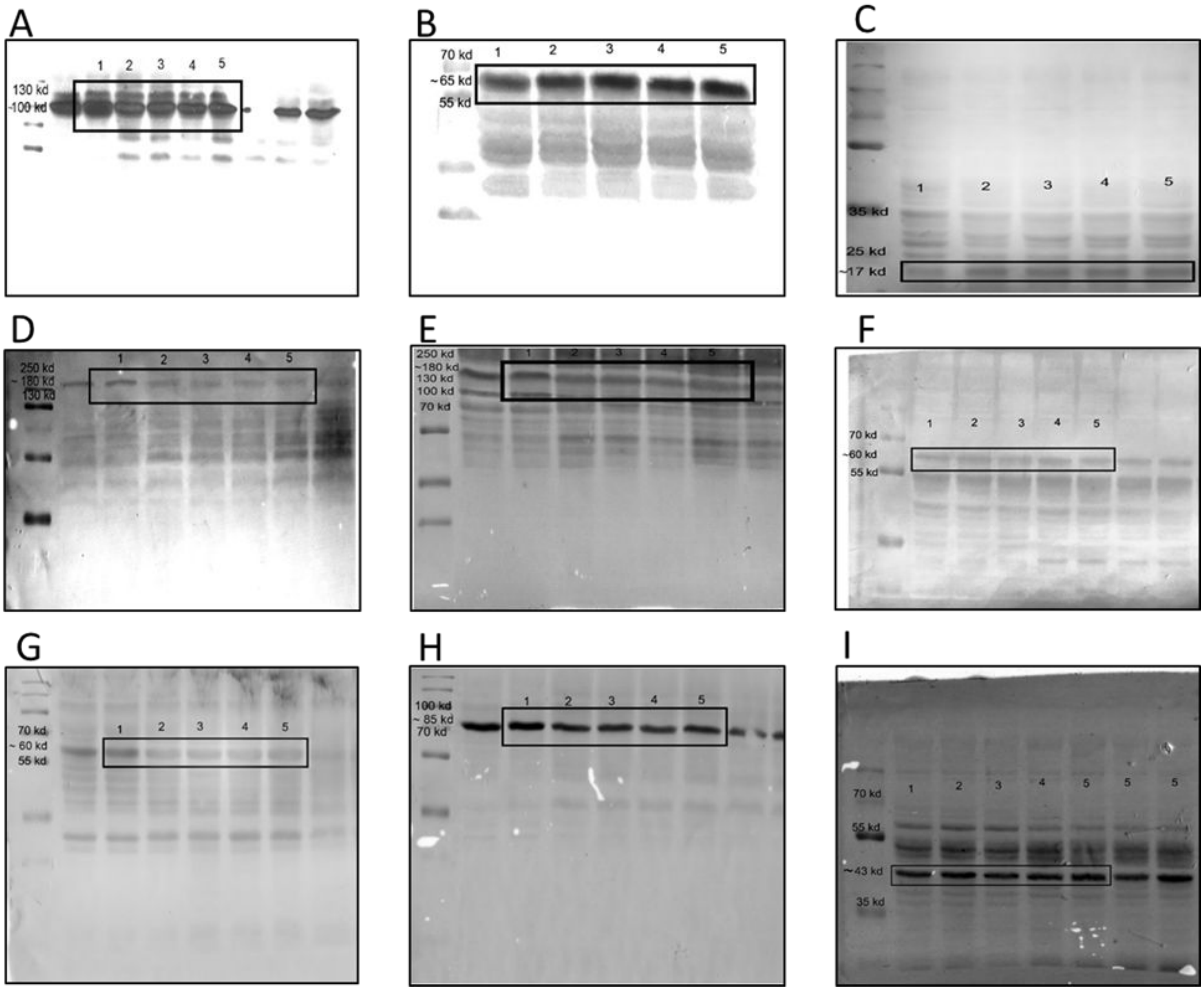

Figure S1. The full blots of liver Nrf2 (A), NF-κB (B), TNF-α (C), total IRS-1 (D), p-IRS-1 (E), total Akt (F), p-Akt (G) and PI3K p85 (H) protein expression levels (J) in rats. β-actin (I) was used as a loading control.

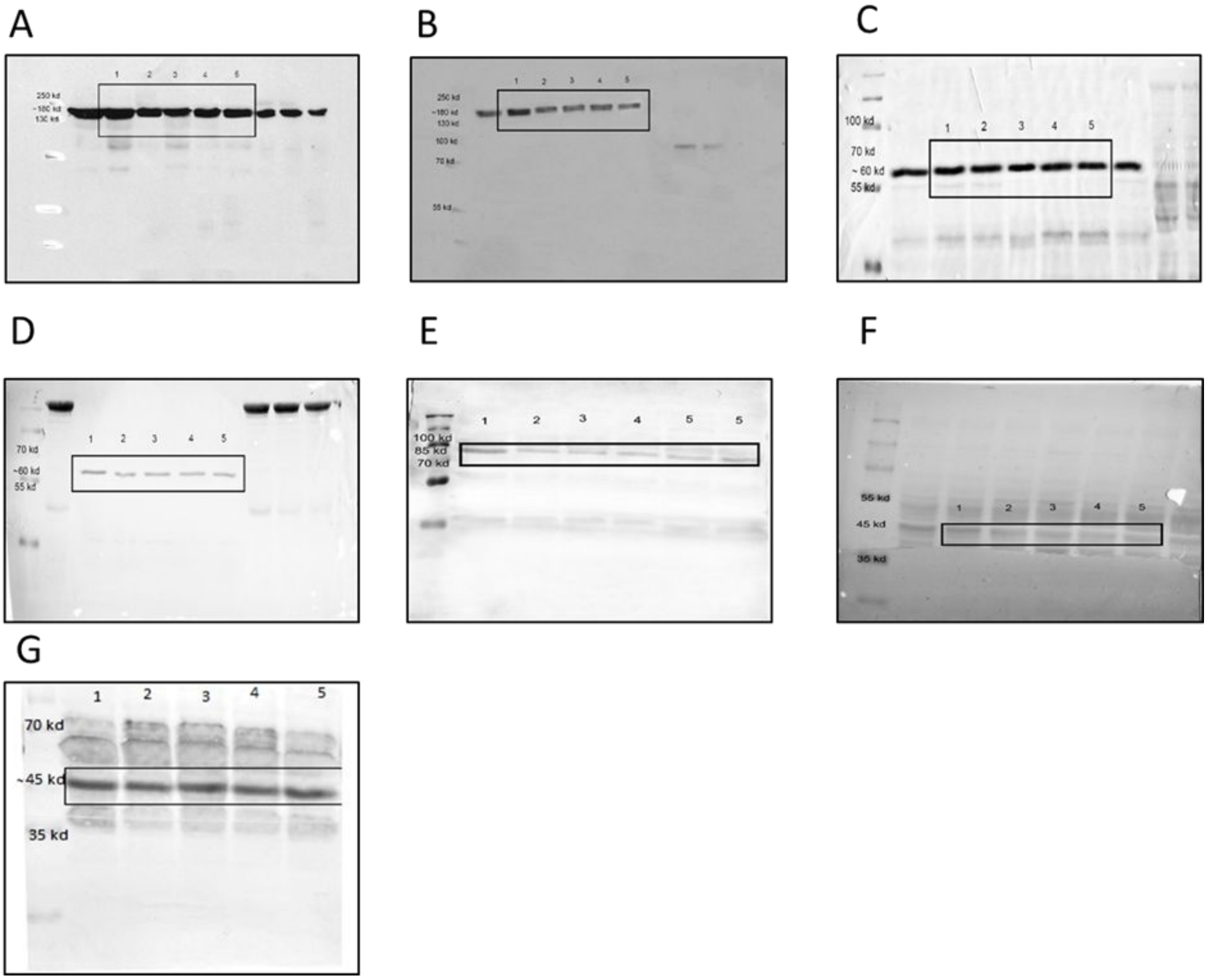

Figure S2. The full blots of muscle total IRS-1 (A), p-IRS-1 (B), total Akt (C), p-Akt (D), PI3K p85 (E), and GLUT4 (F) protein expression levels. β-actin (G) was used as a loading control.
